# An Integrated Individual Environmental Exposure Assessment System for Real-Time Mobile Sensing in Environmental Health Studies

**DOI:** 10.3390/s21124039

**Published:** 2021-06-11

**Authors:** Jue Wang, Lirong Kou, Mei-Po Kwan, Rebecca Marie Shakespeare, Kangjae Lee, Yoo Min Park

**Affiliations:** 1 Department of Geography, Geomatics and Environment, University of Toronto Mississauga, Mississauga, ON L5L 1C6, Canada; gis.wang@utoronto.ca; 2 Department of Geography and Planning, University of Toronto, Toronto, ON M5S 1A1, Canada; 3 School of Tourism Management, Sun Yat-sen University, Guangzhou 510275, China; koulr3@mail.sysu.edu.cn; 4 Department of Geography and Resource Management and Institute of Space and Earth Information Science, The Chinese University of Hong Kong, Shatin, Hong Kong, China; 5 Department of Human Geography and Spatial Planning, Utrecht University, 3584 CB Utrecht, The Netherlands; 6 Department of Urban and Environmental Policy and Planning, Tufts University, Medford, MA 02155, USA; rebecca.shakespeare@tufts.edu; 7 School of Civil and Environmental Engineering, Yonsei University, Seoul 03722, Korea; kkooring@uos.ac.kr; 8 Department of Geography, Planning, and Environment, East Carolina University, Greenville, NC 27858, USA; parky19@ecu.edu

**Keywords:** individual environmental exposure, portable sensors, environmental health, health geography

## Abstract

The effects of environmental exposure on human health have been widely explored by scholars in health geography for decades. However, recent advances in geospatial technologies, especially the development of mobile approaches to collecting real-time and high-resolution individual data, have enabled sophisticated methods for assessing people’s environmental exposure. This study proposes an individual environmental exposure assessment system (IEEAS) that integrates objective real-time monitoring devices and subjective sensing tools to provide a composite way for individual-based environmental exposure data collection. With field test data collected in Chicago and Beijing, we illustrate and discuss the advantages of the proposed IEEAS and the composite analysis that could be applied. Data collected with the proposed IEEAS yield relatively accurate measurements of individual exposure in a composite way, and offer new opportunities for developing more sophisticated ways to measure individual environmental exposure. With the capability to consider both the variations in environmental risks and human mobility in high spatial and temporal resolutions, the IEEAS also helps mitigate some uncertainties in environmental exposure assessment and thus enables a better understanding of the relationship between individual environmental exposure and health outcomes.

## 1. Introduction

The effects of environmental exposure on human health have been investigated by scholars for decades. There is growing evidence indicating the association between environmental exposure and health outcomes or behaviors: obesity and cardiovascular diseases are correlated with the food environment [1,2]; health-promoting physical activity was affected by green space [3,4]; certain environmental exposures could lead to tobacco and substance drug use [5,6,7]; stress, depression and other mental health disorders are related to neighborhood contexts [8,9]. However, the associations between environmental exposures and health were often found to be inconsistent [10,11,12].

Recent studies have identified several fundamental problems that could contribute to such inconsistency [13,14,15]. Among the limitations of past studies is their tendency to adopt a static approach to assessing human exposure to environmental risks by assuming that residential neighborhoods are the most relevant contextual areas that affect individual exposure [10,16]. However, residential neighborhoods may not accurately capture the true geographic contexts that exert influences on people’s health because people move around to perform daily activities and thus are also exposed to environmental risks in non-residential neighborhoods [17,18]. In addition, the temporal dynamics of environmental risks add to the complexity of environmental exposure assessment. For instance, studies have shown that using hourly air pollution concentrations improves the accuracy in assessments of the relationships between environmental exposure and health outcomes, compared with using daily averages of air pollution concentrations [19].

Further, the neglect of human mobility and the temporal variations of environmental risks can lead to erroneous results due to two methodological problems: the uncertain geographic context problem (UGCoP) and the neighborhood effect averaging problem (NEAP) [16,20]. The UGCoP, involving both spatial and temporal uncertainties, arises because of our limited knowledge of the true geographic contexts for assessing their health effects. Spatial uncertainties are due to the imprecise spatial configuration of the contextual areas that influence human health. Temporal uncertainties arise because the timing and duration in which individuals are exposed to environmental risks are ignored. The UGCoP may have confounding effects on the relationships between environmental exposure and health outcomes, leading to inferential errors. The NEAP refers to the tendency of individual mobility-dependent exposures (e.g., noise or air pollution) towards the mean value of the participants or population of a study area when people’s daily mobility is ignored. Because of the NEAP, the health impacts of individual environmental exposures observed with traditional residence-based assessments may be erroneous [11,16].

To mitigate the UGCoP and the NEAP, recent studies have increasingly adopted a dynamic approach to assessing individual environmental exposure [11,21,22], which is individual-based and person-specific, incorporating human mobility and spatiotemporal variations in environmental risks [21,22]. It is based on the premise that exposure to environmental risks is inextricably related to human movement patterns at various spatial and temporal scales. It considers not just where people live but also where they visit and when and how much time they spend at each activity location [20]. Thus, the measurement of individual environmental exposure may need to shift from residential neighborhoods to include multiple geographic contexts of individuals’ everyday life [19] for better environmental exposure assessment.

Advances in geospatial technologies, especially the development of mobile approaches to collecting real-time and high-resolution individual data, have enabled many new methods for examining the health effects of environmental exposure [4,15,23,24,25]. Most of these innovative approaches involve the use of GPS tracking and portable or mobile sensing (e.g., noise and air pollutant sensors). Employing these objective exposure monitoring methods would not only provide more accurate assessments of individual exposure but also help address the major methodological issues and limitations of past studies [19,26]. However, focusing only on objective environmental exposure may ignore the differences between people’s perceptions of the same objective environment. For example, differences in individuals’ perceived exposure to noise and greenspace could lead to variations in individual responses to the same levels of environmental exposure.

Further, different environmental factors may interact, and these interactions may have complex health impacts that may be different from those of each individual factor or those from adding the separate effects of these individual factors. Environmental factors influence human health through complex interactions and mediating pathways that may render their individual impact on human health difficult to ascertain. For instance, green space exposure may increase people’s physical activity and mitigate pollution levels by removing pollutants from the air, while green space is also a source of pollen and may aggravate allergies and increase particulate matter counts. We not only need to measure people’s exposure to individual environmental factors over space and time accurately but also need to consider their complex interactions and the mediating pathways. In another example, it is possible that exposure to high noise levels and high pollution levels simultaneously could aggravate their individual health impacts. Thus it is necessary to integrate objective environmental exposure monitoring and subjective environmental exposure sensing for individual environmental exposure assessment in environmental health studies.

This paper proposes an individual environmental exposure assessment system (IEEAS) that integrates objective environmental exposure monitoring devices (such as portable GPS trackers, air pollution sensors, and noise sensors) and subjective environmental exposure sensing tools (such as ecological momentary assessment) to provide a thorough picture of individual exposure. The integration of these real-time data related to human mobility and experience provides fine-grained, spatially, and temporally situated data about environmental contexts and individual activities and emotions. The IEEAS was first implemented in an exploratory pilot project in Chicago with a small sample (45 participants). The focus of this paper is on implementing the IEEAS and seeing how well it works. The primary purpose of the paper is thus not to present a full-scale research project (e.g., reporting research results in detail) but to illustrate how the system works based on this pilot project. Subsequently, the IEEAS developed in the Chicago pilot project was used in Beijing, China, to collect a second dataset from 120 participants. The paper thus draws from both the Chicago and Beijing projects to illustrate the kind of findings that can be obtained from using the IEEAS and the potential of the IEEAS for individual environmental exposure and health studies. We discuss several selected results to illustrate the advantages of the proposed IEEAS and the possible composite analysis. Both the Chicago project and the Beijing project, although exploratory in nature, are among the few studies to date that simultaneously collected real-time data on people’s exposures to two environmental risk factors (i.e., air pollution and noise). The proposed assessment system presents new opportunities for deploying more sophisticated methods for measuring individual environmental exposure and thus promotes a better understanding of the relationship between individual exposure and health outcomes.

## 2. Materials and Methods

**Figure 1:**
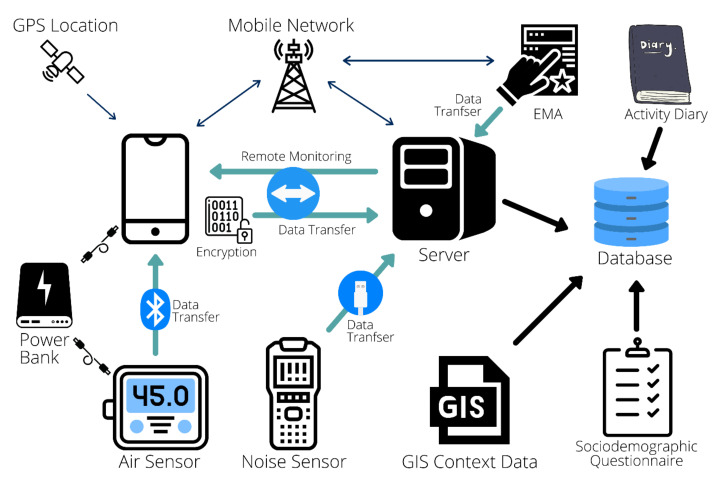
The system overview of the individual environmental exposure assessment system.

This paper proposes an integrated individual environmental exposure assessment system (IEEAS) that combines data from objective environmental exposure monitoring devices and subjective environmental exposure sensing tools. Figure 1 illustrates these key components of the IEEAS, additional sociodemographic information, environmental context GIS data, as well as device monitoring, data encryption, and data transfer.

### 2.1. Integrated Objective Environmental Exposure Monitoring Devices

The development of Global Positioning System (GPS) technologies and portable or mobile environmental sensors provides affordable ways for assessing individual real-time movement patterns and environmental exposure [27,28]. GPS technologies enable researchers to collect high-resolution spatiotemporal data of individuals’ locations and travel routes [29,30]. With their recent advances, GPS and portable environmental sensors are increasingly integrated and used together in recent research to collect high-resolution spatiotemporal data of person-specific real-time environmental exposure. Examples of integrated sensor systems include N-Smart [31], Common Sense [32], MobGeoSen [33].

Although progress has been made in assessing individual-level environmental exposure, several issues still need to be addressed. First, existing studies have mainly focused on air pollution, with limited consideration of other environmental factors such as noise exposure. However, noise and air pollution may be closely related to similar sources and health impacts [34,35]. Further, exposure to air and noise pollution may have similar health effects on the human neurocognitive and cardiovascular systems [36,37,38]. Thus, considering only noise or air pollution may lead to misleading results in the relationships between environmental exposure and its health effects [34,36]. As sensor technologies for more accurate assessment of individual exposure to noise and air pollution become available, more studies are needed to further explore the synergistic relations between real-time noise and air exposure and the mechanisms of how they interact to impact human health.

**Figure 2:**
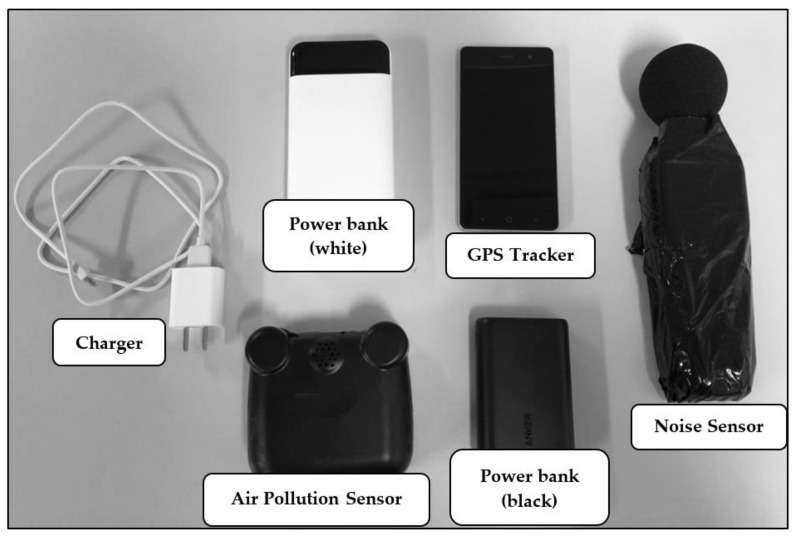
The GPS and portable sensing devices used in the study.

In our pilot study conducted in Chicago, the integrated objective environmental exposure monitoring devices combine a GPS-equipped mobile phone, an air pollution sensor, and a noise sensor, with two additional power banks to provide extra battery life for the portable devices (Figure 2). The lightweight mobile phone served as a hub for data collection and is equipped with a GPS to collect real-time location data. The air pollution sensor was connected to the mobile phone via Bluetooth and transferred real-time air quality data to the phone’s internal memory, while the mobile devices could be remotely accessed using the TeamViewer app, which helped the research team to monitor the status of both the GPS and the air pollution sensor (including battery usage and data collection status). When issues were discovered during remote monitoring, the research team could fix the problems remotely or call the participants to help solve the issues in real-time to avoid data loss as much as possible. The portable noise sensor ran independently and had enough internal memory to store the noise data for the entire data collection period.

The configuration and settings of these portable devices are shown in Table 1. AirBeam (HabitatMap, Brooklyn, NY, USA) was selected as the portable air pollution sensor because of its light weight (198.5 g), long battery life (10 h if fully charged), and reasonable accuracy and precision of its PM_2.5_ measurements. The performance of AirBeam has been evaluated in many previous studies. In the field test performed by the Air Quality Sensor Performance Evaluation Center (AQ-SPEC) in the South Coast Air Quality Management District (SCAQMD, Diamond Bar, CA, USA), the measurements of PM_2.5_ (24-h mean) strongly correlated with Federal Equivalency Method (FEM) GRIMM monitor data (R^2^ > 0.89) [39]. Mukherjee et al. [40] compared three units of AirBeam against the GRIMM 11-R reference instrument and found that the measurements from AirBeam presented robust sensor-to-sensor precision (R^2^ = 0.95–0.99) and moderate agreement with the reference measurements (R^2^ = 0.66–0.71) over a range of different temperature and relative humidity at Cuyama Valley (CA, USA). Similar results were reported in a more recent study that deployed AirBeam at two sites in Sacramento (CA, USA) [41]. SLM-25 portable data-logging sound level meters were used for noise measurement in the study. The portable noise sensors logged the every-minute A-weighted decibels (dBA) of each participant’s surrounding noise. It meets IEC61672 Type 2 and ANSI S1.4 Type 2 Sound Level Meter Standards (accuracy: +1.5 dB; measurement range: 30–130 dBA). To ensure accuracy, each noise sensor was calibrated using a CEM SC-05 Sound Level Calibrator at both C-weighted and A-weighted 94 dB and 114 dB before distribution to each participant.

**Figure 3:**
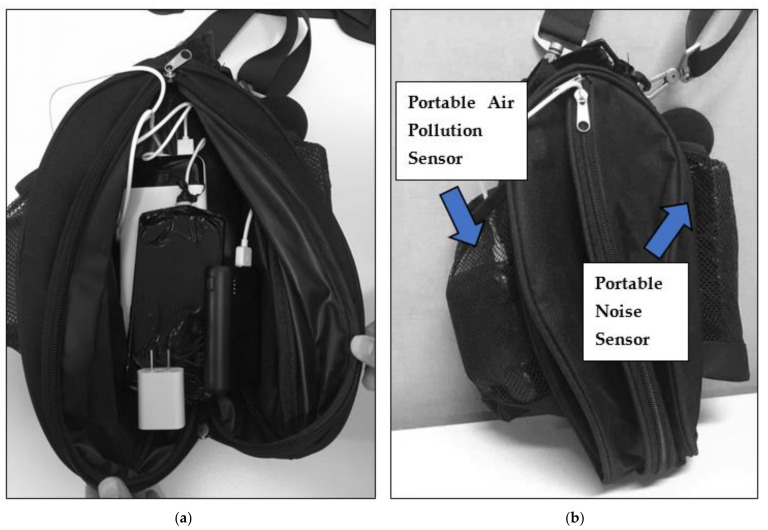
The device bag for the GPS and mobile sensors. (**a**) The portable devices are packed into a device bag; (**b**) air pollution sensors and noise sensors are placed in the two external mesh pockets on the two sides of the bag.

The mobile phone (ZTE A603) worked as the data hub for the GPS and air pollution data, where the GPS trajectory data were recorded by GPS Logger (an Android application), and the air pollution data were stored using AirCasting (an Android application). It is equipped with a GPS with A-GPS support for accurate and reliable location tracking. The device’s 16 GB memory is capable of storing one-week long’s GPS trajectory and air pollution data. The mobile phone worked with TeamViewer (an Android application and desktop software) for the remote monitoring of the status of data collection and data uploading. The two extra power banks provided extra battery life for both the mobile phone and AirBeam to avoid data loss caused by an unexpected battery drain. For ease of use, we packed all the portable devices and the extra power banks in a device bag, as shown in Figure 3a. The air pollution sensors and noise sensors were installed in the two mesh pockets on the two sides of the bag (see Figure 3b) for good air ventilation and the prevention of sound blocking. The size of the mesh cell is relatively large, and we made sure the inlet was facing out for good air ventilation. The participants were asked to carry the bag all the time during the survey days.

In order to maintain the quality of data and minimize the chance of data loss due to technical issues, we utilize TeamViewer to monitor the data collection devices. The status of the devices and the data collection apps were checked through remote access with TeamViewer every 2 h (from 8 a.m. to 10 p.m. every day) by members of the research team to make sure that all the devices are working well. When any device was found to have low battery power, the research team would either call or text the participants to recharge the device. If any device was found to be disconnected, the research team would call the participants to fix the problem.

All data collected by the mobile phone, including GPS tracking and air pollution data, were encrypted in the phone using the open-source data encryption software Secret Space Encryptor (S.S.E.) for Android through remote control by TeamViewer. After the encryption, the data were transferred to the host server using the File transfer function of TeamViewer, while the raw data on the mobile phone was securely shredded. All the encrypted data were finally stored in one external hard disk (encrypted with BitLocker) and backed up in another external hard disk (encrypted with BitLocker). All data were not decrypted until they had been securely transferred to an encrypted (by BitLocker) and password-protected computer with no Internet access in a secure and locked room.

### 2.2. Integrated Subjective Environmental Exposure Sensing Tool

In addition to the objective data collected by GPS trackers and portable sensors, subjective exposures from participants were collected to understand the differences in perceived exposures among people with activity dairy and ecological momentary assessment data. Further, collecting and using data on perceived exposures allows for the triangulation and validation of data collected by multiple methods. This would help provide more accurate and reliable knowledge of the relationships between individuals’ environmental exposure and its health impacts [42,43].

In this study, all participants attended a briefing session at the collaborating organization. During the session, participants (1) went through the consent process, (2) completed the questionnaire, (3) were also trained on how to fill out the activity diary, including how to log specific events and trips, (4) were briefed on how ecological momentary assessment (EMA) text messages would appear in the mobile phones given to them and how to complete them promptly.

Activity diaries were an additional data source to assess individual exposure to environmental risks. They were a useful supplementary data source besides GPS trajectories [43]. Time geographers have frequently used the activity diary method to understand how individuals undertake their daily activities and trips within certain space-time constraints [44]. The activity diary method collects more contextual information about people’s activities and trips [45,46] and allows for more detailed delineations of people’s microenvironments [37,45]. For instance, it can capture the details of different microenvironments such as residential locations, workplaces, recreational places, and transportation environments [47].

In this study, activity diaries were used to collect detailed information of each participant’s daily activities and trips in sequence. Participants completed a 2-day activity diary (one on a weekend day and one on a weekday), in which they briefly logged each activity or trip they performed. For each activity and trip, participants were asked to record the start time, type of companion, space-time fixity (by rating whether the start time and the location can be changed easily), and their subjective evaluation of noise (by rating whether noise is a problem). In addition, for each activity, participants also logged the locations (whether the activity was at home, workplace, or other types of places) and the street addresses of all the locations recorded in their activity diaries, indoor/outdoor (whether the activity was undertaken indoor or outdoor), and the type of the activity. Activity type covers five categories, including work/study, personal affairs, housework, shopping, and recreation, together with other sub-categories in each category. Participants also reported their primary travel mode for each trip, including walking, biking, motorcycle, taxi, bus, subway, shuttle, car, and others. The data collected by the activity diaries were used to investigate the context of each activity. Further, the data collected by the activity diaries (e.g., start time, duration, the address of each activity location, and indoor/outdoor) were used to cross-validate the GPS trajectories.

Ecological momentary assessment (EMA) was another important component in our data collection effort to provide contextualized information. Previous studies have utilized EMA methods to examine issues such as people’s physical activities, their situated physical and social contexts [48,49], emotional experiences such as moods, stress, and anxiety [50], environmental stressors [51], and health-related behaviors and symptoms as they occur in real-time [52,53]. Specifically, EMA helps provide real-time measurements of individuals’ behavioral and psychological states in their natural environments [53]. In addition, repeated assessments in EMA can show fluctuations in environmental exposure over certain periods [51], which helps researchers understand how health-related behaviors and outcomes change over time and across contexts [54]. Further, the collection of EMA data together with GPS data [20,55] not only enriches the EMA data with spatial information but also generates more contextualized information for the GPS data, providing insight into the complex relationships between environmental exposure and health outcomes.

In the Chicago project, EMA was used to collect real-time data of participants’ environmental perceptions and health-related psychological stress. The data were collected with SurveySignal and SurveyMonkey. The configuration of the EMA collection software is provided in Table 2. Participants were requested to answer three short EMA questions: “How much does noise bother or disturb your current activity/trip?”, “How much does air pollution bother or disturb your current activity/trip?”, and “How stressed are you now?”. Responses to each of these questions were provided on a 4-point scale: “not at all”, “slightly”, “moderately”, and “extremely stressed”. This project conducted a time-based EMA survey by sending survey messages to participants at 9 a.m., 12 p.m., 4 p.m., and 8 p.m., respectively, representing the morning, noon, afternoon, and evening of a day. Based on the specific time point of each EMA response from each participant, the contextualized data collected by the activity diaries and the spatiotemporal data collected by GPS were integrated for momentary environmental health analysis.

### 2.3. Additional Sociodemographic Information and Environmental Context GIS Data

Sociodemographic information was collected through questionnaires. During the briefing session, participants were guided to finish a 10-page long questionnaire. The questionnaire includes sections that collect participants’ sociodemographic information, including personal, household, transportation, moving history, community, health, and meals and fitness habits. The personal and household sections contain questions about gender, age, race, education, marital status, employment, income, family size, and dependents. The transportation section provides general information on the ownership of the automobile and commuting habits. The moving history and community sections render a thorough depiction of subjects’ historical and current living environments, comprising questions about their residence locations, conditions, and ownership, as well as the general information and their perception of the neighborhoods. In the health section, questions about their historical and current physical health issues and mental health conditions were included to provide relevant health-related information. The comprehensive information collected in the questionnaire, integrated with the objective and subjective environmental exposure sensing data, provided the health outcome variables for the composite analysis in environmental health studies.

GIS context data is critical for environmental health studies. It could serve as a source of background environmental context information, such as locations of green space and blue space, or as additional data for validation or triangulation with the objective exposure information, such as map matching GPS trajectories with road network data to improve GPS data quality. The needed contextual or environmental data depends on research questions. In this study, focusing on chronic and mental health issues, we collected GIS-based data on road networks, census, community survey, points of interest, land use, air pollution monitoring, transportation noise data, and so on. The environmental context GIS data were obtained from the national census bureau (e.g., US Census Bureau), local authority data portal (e.g., Chicago Data Portal), open-source GIS database (e.g., the OpenStreetMap.com), and other publicly available environmental data (e.g., the national transportation noise map).

### 2.4. Data Collection in the Chicago Pilot Project

We conducted an exploratory study to implement the proposed integrated individual environmental exposure assessment system (IEEAS). It was conducted in the Humboldt Park community of the Chicago Metropolitan Area from October to December 2017 (the Chicago dataset). This suburban community is undergoing a process of gentrification as middle-class residents move into it, while there is still a significant number of low-income residents in the community who live in affordable public housing. We recruited 45 participants in the community with the assistance of the Latin United Community Housing Association (LUCHA), a non-profit organization that provides affordable housing for low-income residents.

All participants attended a briefing session to receive training on how to fill out the surveys and how to carry and use the portable sensors properly. Data collection lasted for a consecutive 7 days, including 5 weekdays and 2 weekend days, for each participant. First, participants completed a personal questionnaire. They also completed an activity diary for two designated survey days (1 weekday and 1 weekend day) in which they logged all activities and trips they performed. Second, participants carried at all times all the tracking and sensing devices in a dedicated device bag provided to them during the 7-day period. To reduce the participants’ burden, all the devices were fully charged before giving them to the participants (the sensors could run for 48 h with a full charge). From Day 3 onwards (after the first 48 h), participants needed to charge all devices at home in the evening before using them the next day. Third, at the end of the 7-day period, participants completed an activity space survey, which recorded the locations they visited during the 7-day period and the locations they regularly visited in a typical week or at least once a week (e.g., workplaces, shopping malls, supermarkets, and restaurants). Note that the results reported later in this paper are based on the 34 participants (from the original 45) who provided a valid home address and have adequate GPS trajectories for implementing the green space and noise exposure assessments. The sociodemographic characteristics of these participants are listed in Table 3. Other contextual or environmental data used in the study were obtained from the Chicago Data Portal, the OpenStreetMap geospatial dataset, and the US Bureau of Transportation Statistics.

The proposed IEEAS had also been implemented to collect a larger dataset in Beijing, China (the Beijing dataset) from December 2017 to February 2018. 120 subjects were recruited from the Meiheyuan community for data collection using the same set-up as the Chicago Project using GPS tracking devices, portable air pollution, and noise sensors, EMA, activity diaries, and questionnaires. Meiheyuan is located in the northeast of Beijing and is a typical inner suburban community. It covers about 91,000 square meters with a total population of about 5000. Table 4 lists the descriptive statistics of the participants’ sociodemographic characteristics.

## 3. Result and Discussion

### 3.1. Exploratory Analysis

Using the Chicago dataset, we conduct some exploratory analysis and discuss successful research that used the proposed IEEAS. Focusing on participants’ exposure to the noise environment, we compare the difference in individual exposures obtained by the proposed system and conventional methods. With the help of the IEEAS, we also examine the interactions among environmental factors and their complex associations with health. Thus, several examples of composite analysis with the data collected based on the IEEAS are further discussed to illustrate its advantages.

#### 3.1.1. Individual Noise Exposure Assessment

People’s noise exposure may vary across space and time in a dynamic and complex manner. Therefore, accurately measuring individual exposures to noise can be challenging. The standard measure of noise exposure is *L_Aeq,24h_*, the A-weighted equivalent continuous sound level over the 24 h of a day [56]. Based on the Chicago dataset, we assessed and compared individual exposure to noise using three measures: home-based *L_Aeq,24h_*, GPS-based *L_Aeq,24h_*, and sensor-based *L_Aeq,24h_*. First, home-based *L_Aeq,24h_* is calculated based on the home location of each participant by overlaying it with the National Transportation Noise Map that models the approximate average noise energy due to traffic-related noise sources. The National Transportation Noise Map is obtained via the geospatial data portal of the Bureau of Transportation Statistics [57]. It was “developed using a 24-h equivalent sound level noise metric as of 19 April 2018” [58], which is very close to the data collection period of the Chicago Project. This is the average noise exposure for an individual based on his/her home location. It is calculated based on a static noise map and thus does not reflect the dynamic noise environment people are exposed to as they undertake their daily activities.

Second, based on participants’ GPS tracking records, the *L_Aeq,24h_* at the locations of each participant’s GPS records are extracted by overlaying them with the national transportation noise map. Then, a time-weighted average of the noise exposure is calculated based on the extracted *L_Aeq,24h_* values at all the GPS records. This is the GPS-based *L_Aeq,24h_*. Compared to the home-based measure, the GPS-based *L_Aeq,24h_* takes into account the spatial dynamics of people’s daily activities and thus provides a better estimation of their overall noise exposure. However, the National Transportation Noise Map used to derive the GPS-based *L_Aeq,24h_* values is still static and cannot incorporate the temporal dynamics of the noise environment.

The third measure of individual exposure to noise in this study is based on the real-time data acquired by the portable noise sensors. It takes into account both the temporal dynamics of the noise environment and the spatial dynamics of people’s daily activities. It is calculated using the noise level data collected from the noise sensors that recorded the different noise levels at different times and locations along with a participant’s daily movement. The formula of *L_Aeq,24h_* is used to calculate the A-weighted equivalent continuous sound level over a period of time T [56]. In this study, the sensor-based accumulated noise exposure *L_Aeq,24h_* is evaluated by considering both a weekday and a weekend day to better represent individuals’ daily noise exposure levels as follows.

(1) $$L_{Aeq,\;T}=10\;\mathrm{lg}(\frac{1}{T}\;\int_{0}^{T}10^{0.1L_{PA}}\;dt)$$

Here, *L_PA_* is the one-minute A-weighted sound level (dBA) (on a logarithmic scale) recorded by the portable noise sensors.

**Figure 4:**
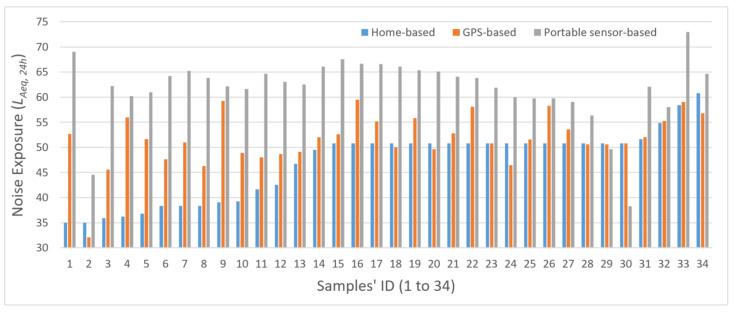
Comparison of individual noise exposure estimated by the three methods (home-based, GPS-based, and portable sensor-based) for each participant.

The noise exposures obtained with these three measures (home-based *L_Aeq,24h_*, GPS-based *L_Aeq,24h_*, and sensor-based *L_Aeq,24h_*) are compared to illustrate their differences and highlight the new opportunities for evaluating individual exposures to dynamic environmental contexts. Figure 4 compares the three individual noise exposure measures: home-based *L_Aeq,24h_*, GPS-based *L_Aeq,24h_*, and sensor-based *L_Aeq,24h_*. In the figure, the horizontal axis represents the 34 participants in this analysis, while the vertical axis shows the values of the three measures of noise exposure. As indicated in the figure, individual noise exposures estimated by the three metrics are significantly different from each other.

The differences in the individual noise exposure estimated by the three methods are further investigated with descriptive statistics and correlation analysis (Table 5), which shows that the sensor-based measure gives relatively high exposure values, while the home-based measure has relatively low assessments among the three methods. The home-based measure has the lowest mean (46.81) and the highest standard deviation (7.11), while the sensor-based measure gives the highest mean (61.71). It is worth noting that the individual noise exposures assessed by the home-based and GPS-based measures are significantly correlated (*p* < 0.01). In addition, the measures obtained by the GPS-based and sensor-based methods are also significantly correlated (*p* = 0.01). However, there is no significant association (*p* > 0.1) in individual noise exposures between the home-based and sensor-based measures.

Table 6 shows the results of the paired sample *t*-test, which compares the pairwise differences in individual noise exposure between the three measures. As shown in the table, all of the three pairs of noise exposure measures show significant differences. For Pair 1 (home-based versus GPS-based measures), noise exposure assessed by the home-based measure is less than the values obtained by the GPS-based measure (*p* < 0.01), and the differences are statistically significant. On average, participants’ home-based exposure levels are 4.9 (95% CI: 2.64, 7.16) units lower than their GPS-based exposure levels. For Pair 2 (home-based versus sensor-based measures), the noise exposures evaluated by the home-based measure are less than the values obtained by the sensor-based measure (*p* < 0.01), and the differences are statistically significant.

On average, the home-based exposure levels are 14.9 *L_Aeq,24h_* (95% CI: 11.67, 18.14) units lower than the sensor-based exposure levels. For Pair 3 (GPS-based versus sensor-based measures), the noise exposures assessed by the GPS-based measure is significantly different from the ones assessed by the sensor-based method (*p* < 0.01) in that the GPS-based exposure levels are 10.00 *L_Aeq,24h_* (95% CI: 7.77, 12.22) units lower than the sensor-based exposure levels on average.

It is clear from these results that individual noise exposures measured by the three methods are significantly different. As Table 5 indicates, the home-based measure has the lowest mean and highest standard deviation. Thus, it significantly underestimated individual exposures to noise. There are two possible reasons for this. First, the home-based measure is an averaged indicator of noise exposure for an individual based only on the person’s home location, so the measure does not consider the spatial dynamics of people’s daily activities. Studies have shown that people move around to perform various daily activities (e.g., work and shopping) and only spend a limited amount of time at home [18,59,60]. The residential neighborhood only partially captures people’s exposure to noise and may thus lead to inaccurate measurement. Second, the home-based *L_Aeq,24h_* is calculated based on the National Transportation Noise Map, which is an approximate average noise energy level based on transportation noise sources over a 24-h period. The noise map is an averaged indicator of ambient noise and is a static representation of noise distribution that does not consider the temporal variations in the noise environment. However, the noise environment is dynamic and constantly changing over time. Ignoring the temporal dynamics of the noise environment when assessing individual noise exposure may introduce bias. Furthermore, the National Transportation Noise Map only takes into account one source of noise (transport-related noise) while ignoring all other sources of noise (e.g., construction activities) that individuals may be exposed to in their daily activities [61].

Because the noise sensors precisely recorded the noise level in each participant’s immediate environment in real-time at a high spatial and temporal resolution, the sensor-based measure gives the most accurate measurement of individual noise exposure. In addition, the noise sensors capture individual noise exposure at multiple locations, in various microenvironments, and at different times, such as sleeping at home at night, working at workplaces during work time, and commuting in the morning and late-afternoon rush hours. Further, the noise sensors can capture multiple sources of noise, including transportation, commercial activities, and construction activities. With the capability to consider both the spatial and temporal variations in noise level as well as human mobility, this method gives the most accurate measurement of individual exposure and thus helps mitigate the UGCoP and the NEAP in noise exposure assessment.

Interestingly, the range of noise exposure obtained by the sensor-based measure for most of the participants is from about 60 to 65 dBA (with relatively small standard deviations), which is much smaller than the one measured by the home-based method (from about 38 to 51 dBA). A possible reason for this may be that the sensor-based measure considers human mobility. Thus, the overall noise exposure level is subject to neighborhood effect averaging (as a result of an averaging of the exposures at different locations for various daily activities).

As a manifestation of the neighborhood effect averaging problem (NEAP), the sensor-based exposure measurements among the participants would thus show smaller variation when compared to the home-based measurements [16]. Since the home-based measure considers only the home location, noise levels may vary significantly depending on the residential location of the participant. Thus, participants whose homes are located near major highways or airports would have much higher noise exposure when compared to those with homes in quiet or remote neighborhoods. Thoroughly considering all noise exposures at different activity locations, the sensor-based method provides a better estimation of individual noise exposure.

#### 3.1.2. Composite Environmental Health Analysis

Integrating objective exposure monitoring and subjective exposure sensing helps us understand the complex pathways of environmental effects on health. For instance, integrating GPS trajectories and activity dairies promotes our understanding of individual mobility and activity space analysis [62]; integrating GPS trajectories, air pollution sensor data, and environmental context data enrich our understanding of individual air pollution exposure and its relationship with environmental contexts as well as health outcomes [22]; integrating GPS trajectories, noise sensor data, and environmental context data enhances our understanding individual noise exposure and its relationship with health [61]. In what follows, we further discuss and highlight the advantages of using composite analysis based on three studies that used the dataset collected in Beijing with the IEEAS (the Beijing dataset).

Ma et al. [22] investigated the relationship between individuals’ socioeconomic status and their ability to avoid air pollution exposure through daily mobility based on the Beijing dataset. In the study, indoor and outdoor air pollution exposure can be distinguished by integrating the data of portable air pollution sensors, GPS tracking, and activity dairies (participants indicated whether activities happened indoor or outdoor). By comparing the air pollution exposures participants’ experienced in indoor or outdoor locations, the study found significant evidence of neighborhood effect averaging in air pollution exposure assessment and explored whether the air pollution exposures of different social groups can be mitigated by changing mobility patterns. The study shows that data collected with the IEEAS can provide a much more accurate and refined picture of individual exposure to air pollution exposure and how the NEAP may be mitigated.

Using the integrated approach of the IEEAS and the Beijing dataset, Kou et al. [61] examined the direct and indirect effects of context, momentary measured noise, and perceived noise on individual psychological stress levels. The study found that momentary measured noise influences psychological stress through the mediating effect of perceived noise, and different activity, travel, social, and temporal contexts significantly influence people’s momentary measured noise, perceived noise, and psychological stress. These findings greatly enhance our understanding of how the relationship between individual noise exposure and psychological health is influenced by perceived noise and context at a high spatiotemporal resolution.

Similarly, Ma et al.’s [63] study assessed individual noise exposure and its relationship with mental health using the Beijing dataset. The study compared the noise exposure assessed by the integrated approach based on the IEEAS with the conventional static residence-based approach. The study explored how individual noise exposures differ among various daily activities and travel modes and how those exposures varied between workdays and weekends. With composite analysis, the research further examined the noise exposure effects on mental health and found that noise exposure in different activity contexts has various associations with individual mental health.

### 3.2. Limitations and Future Direction

The Chicago and Beijing Projects reported in this paper have several limitations that should be addressed in future research. First, the sample sizes are relatively small. Collecting data from larger samples is necessary to provide further evidence on the relative strengths of different exposure measurement methods. Second, the data were collected from one urban community in Chicago and Beijing separately, which limits the generalizability of the framework to other locations with different social and physical environments (e.g., rural communities). Future studies should be conducted in various cities or countries that cover larger areas. Third, we used the datasets to perform only limited analysis. However, more studies using other integrated approaches are necessary for thoroughly exploring this kind of integrated system and generating new insights. Additionally, new analytical approaches should be developed based on the proposed IEEAS in future environmental health studies. Fourth, although the air pollution sensors used in this study were factory-calibrated, they were not calibrated in the Chicago Pilot Project before its deployment. Because sensor measurements can be biased due to relative humidity and temperature, future studies should evaluate the meteorological impacts and adjust raw measurements using correction algorithms [64]. We chose AirBeam1 because it has reasonable performance according to the literature and has important features needed for the project, such as portability and reasonable price. In addition, when this study was performed, AirBeam1 was the most widely used portable sensor that had several evaluation results. However, AirBeam1 is only one example that can be integrated into the IEEAS. Therefore, it can be replaced with a newer version (AirBeam2) or other portable air sensors that provide higher accuracy, precision, and reliability depending on the study purpose. Fifth, the accuracy of the GPS tracking data may be constrained by the tracking device used in this study. In addition, accuracy may vary from location to location due to the urban canyon effect. Nevertheless, in the Chicago Project, tracking inaccuracy due to urban canyons in the study area is minimal because it is a suburban area in Chicago where all buildings are low and the streets are wide. In addition, GPS tracking devices can be easily replaced with the latest version that provides higher accuracy depending on the specific need of study. Lastly, we installed the air pollution sensor in a side mesh pocket, which may result in potential PM_2.5_ sample loss. Although the inlet was facing out for good air ventilation when setting up the device in a mesh pocket, in future studies, it would be better to set up the inlet of the air sensor without any obstruction to minimize this potential issue. Note that it is possible to integrate other portable devices into the IEEAS for more comprehensive exposure assessments. Other mobile devices such as accelerometers, thermometers, hygrometers, and heart rate monitors could be integrated into the system. Future studies are necessary to investigate the feasibility of integrating these other portable devices for environmental exposure assessment.

## 4. Conclusions

In this study, we proposed an individual environmental exposure assessment system (IEEAS) that integrates the objective monitoring devices and subjective sensing tools, as well as sociodemographic information and environmental context GIS data, to provide a composite way for individual-based environmental exposure data collection for environmental health studies. The IEEAS integrated objective and subjective data collected in real-time provide fine-grained, spatially, and temporally situated data about the measured environmental context, personal activity, and individual perception. The key measurements in the data collection process include the levels of noise or air pollutants (PM_2.5_) in real-time as measured by the portable sensors, the subjective assessments of the levels of noise or air pollutants, and the levels of stress reported by the participants via the EMA prompts received on their mobile phones, and details of participants’ activities and trips on the survey days (e.g., the start time, end time and duration of each activity and trip, the address of each activity location, and whether an activity was conducted in an indoor or outdoor environment) recorded by the activity diaries, which in turn can be used to cross-validate the GPS trajectories. In addition, data in the activity diaries can be used to identify participants’ microenvironments (e.g., workplaces, shopping malls, supermarkets, and restaurants).

This integrated assessment system enables linking empirically measured or sensed environmental characteristics with participants’ activities and momentary experiences to validate, differentiate, and augment sensed data with actual activities and emotional responses to more clearly understand the interrelatedness of measured and experienced real-time environments. Further, the IEEAS enables researchers to capture accurate individual exposure to environmental risks in different microenvironments, involving the risks from diverse sources, at different geographic locations and times, and in both the outdoor and indoor environments. With the capability to consider both the spatial and temporal variations in environmental risks as well as human mobility, the IEEAS also helps mitigate the UGCoP and the NEAP in environmental exposure assessment. It yields relatively accurate measurements of individual exposure in a composite way and presents new opportunities for developing more sophisticated ways to measure individual environmental exposure, and thus promotes a better understanding of the relationship between individual environmental exposure and health outcomes.

## Figures and Tables

**Table 1:** The configuration and settings of the portable devices and software.

Devices & Software	Configuration	Data Collection
Portable air pollution sensors	AirBeam (PM2.5 in the unit of µg/m^3^; 2000 mAh)	Air pollution data collection
Portable noise sensors	SLM-25 (accuracy: +1.5 dB; measurement range: 30–130 dBA)	Noise data collection
Mobile phone	ZTE A603 (GPS with A-GPS support; 16 GB memory; 2400 mAh)	Hub for GPS and air pollution data collection and storage
Power bank (white)	Aigo E20000+ (20,000 mAh)	Provide extra battery life for the air pollution sensor
Power bank (black)	Anker PowerCore (10,000 mAh)	Provide extra battery life for the mobile phone
GPS Logger	Android application (installed on the mobile phone)	GPS data collection.
TeamViewer	Android application & Desktop software (installed in the cellphone and remote monitor computers)	Remote monitoring of the data collection
AirCasting	Android application (installed in the cellphone)	Communicating with the air pollution sensor
Secret Space Encryptor	Android application & Desktop software (installed in the mobile phone and remote monitor computers)	Encrypting the GPS trajectory and air pollution data in the mobile phone to protect participants’ privacy

**Table 2:** The configuration of the ecological momentary assessment (EMA) collection software.

Devices and Software	Description	Data Collection
SurveySignal	Survey distribution application	Sending out time-based EMA texts with links of the survey to participant’s mobile phone
SurveyMonkey	Online survey tools	Collecting and management EMA survey results

**Table 3:** Descriptive statistics of the Chicago participants’ sociodemographic characteristics.

Sociodemographic Variables		Proportion
Gender	Female	38.7%
	Male	61.3%
Race	White	10%
	African American	42%
	Latino/Hispanic	42%
	Other	6%
Education	Elementary School	7%
	High School	58%
	College/University	32%
	Graduate School	3%
Maritial Status	Married	18%
	Others	82%
Annual Income (USD)	Less than 10,000	58%
	10,000–24,999	19%
	25,000–49,999	10%
	50,000–99,999	10%
	100,000 or more	3%

**Table 4:** Descriptive statistics of the Beijing participants’ sociodemographic characteristics.

Sociodemographic Variables		Proportion
Gender	Female	49%
	Male	51%
Employment	Employed	77%
	Unemployed	23%
Maritial Status	Married	68%
	Others	32%
Annual Income (RMB)	Less than 180,000	13%
	180,000–539,999	49%
	540,000–101,900	25%
	102,000 or more	13%

**Table 5:** Descriptive statistics and correlation between the three measures of individual noise exposure.

		Mean	Std. Deviation	Std. Error Mean	Correlation	*p*-Value
Pair 1	Home *L_Aeq,24h_*	46.81	7.11	1.22	0.48	<0.01
	GPS *L_Aeq,24h_*	51.71	5.20	0.89		
Pair 2	Home *L_Aeq,24h_*	46.81	7.11	1.22	0.08	0.64
	Portable *L_Aeq,24h_*	61.71	6.59	1.13		
Pair 3	GPS *L_Aeq,24h_*	51.71	5.20	0.89	0.44	0.01
	Portable *L_Aeq,24h_*	61.71	6.59	1.13		

Notes: *N* = 34; Home *L_Aeq,24h_*: home-based individual noise exposure assessment metric; GPS *L_Aeq,24h_*: GPS trajectory-based individual noise exposure assessment metric; Portable *L_Aeq,24h_*: portable sensor based individual noise exposure assessment metric.

**Table 6:** Paired sample *t*-test between the three measures of individual noise exposure.

		Paired Differences					t	df	*p*-Value
		Mean	Std. Deviation	Std. Error Mean	95% Confidence Interval of the Difference				
					Lower	Upper			
Pair 1	Home *L_Aeq,24h_* &GPS *L_Aeq,24h_*	−4.90	6.48	1.11	−7.16	−2.64	−4.41	33	<0.01
Pair 2	Home *L_Aeq,24h_* & Portable *L_Aeq,24h_*	−14.90	9.28	1.59	−18.14	−11.67	−9.36	33	<0.01
Pair 3	GPS *L_Aeq,24h_* & Portable *L_Aeq,24h_*	−10.00	6.37	1.09	−12.22	−7.77	−9.15	33	<0.01

## Data Availability

The data collected in the study cannot be shared because there is no feasible method for sharing the high-resolution georeferenced (e.g., GPS) data collected without compromising the participants’ privacy.
